# Inference of radio-responsive gene regulatory networks using the graphical lasso algorithm

**DOI:** 10.1186/1471-2105-15-S7-S5

**Published:** 2014-05-28

**Authors:** Jung Hun Oh, Joseph O Deasy

**Affiliations:** 1 Department of Medical Physics, Memorial Sloan-Kettering Cancer Center, New York, NY, USA

## Abstract

**Background:**

Inference of gene regulatory networks (GRNs) from gene microarray expression data is of great interest and remains a challenging task in systems biology. Despite many efforts to develop efficient computational methods, the successful modeling of GRNs thus far has been quite limited. To tackle this problem, we propose a novel framework to reconstruct radio-responsive GRNs based on the graphical lasso algorithm. In our attempt to study radiosensitivity, we reviewed the literature and analyzed two publicly available gene microarray datasets. The graphical lasso algorithm was applied to expression measurements for genes commonly found to be significant in these different analyses.

**Results:**

Assuming that a protein-protein interaction network obtained from a reliable pathway database is a gold-standard network, a comparison between the networks estimated by the graphical lasso algorithm and the gold-standard network was performed. Statistically significant *p*-values were achieved when comparing the gold-standard network with networks estimated from one microarray dataset and when comparing the networks estimated from two microarray datasets.

**Conclusion:**

Our results show the potential to identify new interactions between genes that are not present in a curated database and GRNs using microarray datasets via the graphical lasso algorithm.

## Background

In recent years, there has been a great interest in identifying radio-responsive genes across the whole genome using gene microarray data in the field of radiation oncology. To develop new biomarkers for radiation exposure, Templin *et al*. used whole genome microarray and miRNA data generated from blood samples of patients who underwent total body irradiation in preparation for stem cell transplantation [1]. Rieger and Chu utilized oligonucleotide microarrays with cell lines collected from 15 healthy individuals to investigate the transcriptional response of 10,000 genes in DNA damage to ionizing radiation (IR) and ultraviolet (UV) radiation [2]. In another study, Rieger *et al*. explored transcriptional responses to radiation in lymphoblastoid cells collected from 57 patients and found 20 IR-responsive and 4 UV radiation-responsive genes predictive of radiation toxicity [3]. Eschrich *et al*. employed systems biology modeling to better understand radiosensitivity by identifying novel radiation-specific biomarkers [4]. With gene expression profiles from 48 human cancer cell lines, this biomarker discovery platform resulted in a key radiosensitivity network with 10 hub genes. In our previous study [5], we identified important radio-responsive genes using literature review and gene microarray data analysis. With a systems biology approach, we found a core radio-responsive protein interaction network and its key biological processes using gene ontology analysis.

Gene regulatory networks (GRNs) provide simplified representations and easy interpretation of biological processes in an organism under given conditions [6]. However, inference of GRNs remains a major challenging problem in systems biology, although a number of approaches have been proposed [7]. Cai *et al*. employed sparse structural equation models (SEMs) that integrate gene expression and *cis*-expression quantitative trait loci data for improving inference accuracy and proposed a systematic inference method for SEM estimation [8]. Based on Bayesian analysis using a non-parametric Gaussian process modeling, a novel method for inferring GRNs was proposed by Aijö and Lähdesmäki [9]. This approach enables the use of both time-series and steady-state gene expression measurements to improve the inference of GRNs. Menéndez *et al*. used a Gaussian Markov Random Field (GMRF) to deal with the problem of reverse engineering in GRNs and applied the graphical lasso algorithm to effectively estimate undirected graphical models [10]. Applying a lasso penalty to the problem of inverse covariance matrix estimation facilitated a fast and efficient calculation. The graphical lasso algorithm, which uses a blockwise coordinate descent approach to estimate a sparse graphical model, was proposed by Friedman *et al*. [11].

In this study, we employed a multi-component filtering process, based on a systems biology approach that was proposed in our previous study [5], that narrows down the list of gene candidates and applied the graphical lasso algorithm to microarray datasets to infer radio-responsive GRNs. To estimate the accuracy of the network modeling, the estimated networks were compared with a reliable protein interaction network produced by a manually curated protein interaction database.

## Methods

### Datasets

In our previous study [5], we attempted to investigate all putative genes implicated in radiation response through literature review using PubMed and Scopus search engines and found a list of 221 genes associated with radiation response. In addition, to identify significant radio-responsive genes, biological processes, and pathways, further analysis was performed using two publicly available microarray datasets (GSE23393 [1] and GSE1977 [2]) downloaded from Gene Expression Omnibus (GEO) database. In GSE1977, lymphoblastoid cells collected from 15 healthy individuals were exposed to 5-Gy radiation doses and harvested 4 hours later. To examine the change in gene expression after IR, only mock and IR cases were used. In GSE23393, blood was obtained from eight radiotherapy patients treated at our institution immediately before irradiation and at 4 hours after total body irradiation with 1.25-Gy X-rays. In this study, to explore GRNs associated with radiation response, we used the resulting information obtained from our literature review and reanalyzed the two microarray datasets.

### Identification of significant genes

In the preprocessing stage, gene expression measurements were log-base-2 transformed, followed by quantile normalization across all samples. In our previous study [5], to identify significant genes that have considerable differential expression between before and after irradiation, we used a permutation test. In this study, we employed Significant Analysis of Microarrays (SAM) that resulted in a false discovery rate (FDR) and fold-change for each gene [12]. To minimize the possibility of omitting interesting genes, significant genes identified in the two different techniques were combined for further analysis.

### Pathway analysis

Significant genes identified in our analysis were entered into a manually curated pathway database (MetaCore™, GeneGo, Inc.). This system leads to the most probable protein interaction network based on a list of genes uploaded by the user. We converted this resulting network to an undirected network and used it as a gold-standard network to assess the GRNs estimated by the graphical lasso algorithm.

### Graphical lasso algorithm

In recent years, increasing attention has been paid to the estimation of sparse inverse covariance using *L*_1 _(lasso) regularization [11,13,14]. This approach has been efficiently applied to the investigation of sparse undirected graphical models. In the graphical model, a node represents a feature (gene or protein in this study) and an edge between two nodes represents the relationship between the two corresponding features. In particular, each nonzero off-diagonal element in the inverse covariance matrix indicates that there is a dependency between the two features. That is, as the number of zero off-diagonal elements in the inverse covariance matrix increases, sparser graphs are yielded.

The underlying assumption behind the graphical lasso is that a data matrix **X***_n_*_×_*_p _*consisting of *p *features measured on *n *observations follows a multivariate Gaussian distribution with mean *μ *and covariance **Σ **[10]. Let $\text{Θ}={\text{Σ}}^{-1}$ be the precision matrix, and let **S **denote the covariance matrix of the data. The problem is to maximize the penalized log-likelihood over nonnegative definite matrices  Θ, taking the form

(1) $$\text{log}\left(\text{det}\left(\text{Θ}\right)\right)-\text{trace}\left(\text{S}\text{Θ}\right)-\lambda ∥\text{Θ}{∥}_{1}$$

where $∥\text{Θ}{∥}_{1}$ is the *L*_1 _norm that is the sum of the absolute values of the elements of ${\text{Σ}}^{-1}$, and  λ is a nonnegative tuning parameter that controls the sparsity of the estimated network. More specifically, large values of  λ lead to sparse networks due to the lasso-type penalty, whereas small values of  λ lead to dense networks. Note that the problem that maximizes the penalized log-likelihood is convex [11]. The subgradient equation for Eq. (1) is

(2) $$\frac{\partial }{\partial \text{Θ}}l\left(\text{Θ}\right)=\text{W}-\text{S}-\lambda \cdot \text{sign}\left(\text{Θ}\right)=0$$

where $\text{W}={\text{Θ}}^{-1}$. The block coordinate descent approach partitions the rows and columns such that the target column is always the last, cycling through all features in turn. The partition of  Θ,  W, and  S is defined as:

$$\text{Θ}=\left(\begin{array}{cccc} \theta_{11} & \cdots \; & \theta_{1p-1} & \theta_{1p}\\ & \vdots & & \vdots \\ \theta_{p-11} & \cdots \; & \theta_{p-1p-1} & \theta_{p-1p}\\ \theta_{1p} & \cdots \; & \theta_{p-1p} & \theta_{pp} \end{array}\right)=\left(\begin{array}{cc} {\text{Θ}}_{11} & \theta_{12}\\ \theta_{12}^{\text{T}} & \theta_{22} \end{array}\right),$$

$$W=\left(\begin{array}{cccc} w_{11} & \cdots \; & w_{1p-1} & w_{1p}\\ & \vdots & & \vdots \\ w_{p-11} & \cdots \; & w_{p-1p-1} & w_{p-1p}\\ w_{1p} & \cdots \; & w_{p-1p} & w_{pp} \end{array}\right)=\left(\begin{array}{cc} W_{11} & w_{12}\\ w_{12}^{\text{T}} & w_{22} \end{array}\right),$$

$$S=\left(\begin{array}{cccc} s_{11} & \cdots \; & s_{1p-1} & s_{1p}\\ & \vdots & & \vdots \\ s_{p-11} & \cdots \; & s_{p-1p-1} & s_{p-1p}\\ s_{1p} & \cdots \; & s_{p-1p} & s_{pp} \end{array}\right)=\left(\begin{array}{cc} S_{11} & s_{12}\\ s_{12}^{\text{T}} & s_{22} \end{array}\right)$$

where the size of ${\text{Θ}}_{11}$, $W_{11}$, and $S_{11}$ is (*p *− 1) × (*p *− 1); the size of $\theta_{12}$, ${\text{w}}_{12}$, and $s_{12}$ is (*p *− 1) × 1; and $\theta_{22}$, $w_{22}$, and $s_{22}$ are scalars. Inspired by this approach, Friedman *et al*. proposed to use a conventional lasso algorithm to solve Eq. (2). Here we describe how to solve Eq. (2). The upper-right block of Eq. (2) is

(3) $${\text{w}}_{12}-{\text{s}}_{12}-\lambda \cdot \text{sign}\left({\text{θ}}_{12}\right)=0.$$

The lower-right block of Eq. (2) is

(4) $$w_{22}-s_{22}-\lambda =0.$$

Since $\text{W}={\text{Θ}}^{-1}$, we have

(5) $$\text{W}\times \text{Θ}=\left(\begin{array}{cc} \text{I} & 0\\ 0^{\text{T}} & 1 \end{array}\right)$$

from which we derive

$${\text{W}}_{11}{\text{θ}}_{12}+{\text{w}}_{12}\theta_{22}=0,$$

$${\text{w}}_{12}{\text{θ}}_{12}+w_{22}\theta_{22}=1.$$

Then, we have

$${\text{θ}}_{12}=-\theta_{22}\text{β},$$

$$\theta_{22}=1/\left(w_{22}-w_{12}^{\text{T}}\text{β}\right)$$

where $\text{β}=W_{11}^{-1}w_{12}$. Since $\theta_{22}>0,$$\text{sign}\left({\text{θ}}_{12}\right)$ in Eq. (3) is equal to $\text{sign}\left(-\theta_{22}\text{β}\right)$ =$-\text{sign}\left(\text{β}\right).$ Therefore, Eq. (3) can be rewritten as

(6) $${\text{W}}_{11}\text{β}-s_{12}+\lambda \cdot \text{sign}\left(\text{β}\right)=0.$$

We note that Eq. (6) is equivalent to the gradient equation of the regular lasso problem. At each partition, a lasso regression is fitted. Then, $w_{12}$ and $w_{22}$ are calculated and inserted into **W **before a new partition is made. This procedure is repeated until **W **is converged. Table 1 summarizes the graphical lasso algorithm.

**Table 1:** Graphical lasso algorithm.

1. Initialize **W **= **S + λI**. Note that the diagonal of **W **is fixed in what follows. 2. Repeat the following steps until **W **is converged. a. Partition the matrix **W **such that the target column is last. b. Solve the lasso problem using the coordinate descent algorithm. c. Update $w_{12}=W_{11}\text{β}.$ 3. In the final cycle, calculate $\theta_{12}=-\beta \theta_{22}$ with $\theta_{22}=1/\left(w_{22}-{\text{w}}_{12}^{\text{T}}\beta \right)$.

### Evaluation of estimated gene regulatory networks

To compare the GRNs estimated using the graphical lasso algorithm with a gold-standard network produced by MetaCore software, we used Recall, Precision, and *f*-score metrics defined as follows [15]:

$$Precision=\frac{\text{TP}}{\text{TP}+\text{FP}},$$

$$Recall=\frac{\text{TP}}{\text{TP}+\text{FN}},$$

$$f-score=\frac{2\times Precision\times Recall}{Precision+Recall}$$

where TP indicates the true positives (correctly inferred edges); FP represents the false positives (edges inferred in the estimated network, but not present in the gold-standard network); and FN signifies the false negatives (edges present in the gold-standard network, but not inferred).

## Results

### Identification of significant genes using microarray datasets

Using SAM, significant genes were identified for the GSE1977 and GSE23393 datasets. For GSE1977, 61 genes were identified, with a cutoff of FDR < 20%, including 44 induced genes and 17 repressed genes. For GSE23393, 64 genes were identified, with a cutoff of FDR < 20%, including 19 induced genes and 45 repressed genes. Note that more genes were induced in GSE1977, whereas more genes were repressed in GSE23393. We examined commonly found genes in the two datasets. Table 2 shows the 21 overlapping genes with their fold-change and FDR. For these genes, considerable fold-changes were observed: averaged fold-changes for induced and repressed genes were 3.02 and 0.53, respectively, in GSE1977 and 3.12 and 0.60, respectively, in GSE23393. There was no significant difference in fold-change between GSE1977 and GSE23393 (*p *= 0.64 using Wilcoxon signed-rank test). It is worthwhile to note that induced genes had relatively lower FDR than repressed genes in both datasets.

**Table 2:** A list of 21 genes commonly identified in both microarray datasets using Significant Analysis of Microarrays.

	Gene Symbol	Entrez Gene ID	GSE1977		GSE23393	
			
			Fold-change	FDR (%)	Fold-change	FDR (%)
Induced Genes	ACTA2	59	2.02	0.00	1.85	0.00
	ATF3	467	3.05	0.00	1.40	9.79
	BAX	581	1.53	0.00	1.80	0.00
	BBC3	27113	4.00	0.00	1.48	9.79
	CCNG1	900	1.72	0.00	1.51	0.00
	CD70	970	2.07	0.00	4.85	0.00
	DDB2	1643	2.38	0.00	5.33	0.00
	EI24	9538	1.92	0.00	1.59	3.20
	FDXR	2232	2.34	0.00	13.12	0.00
	GADD45A	1647	4.50	0.00	2.34	0.00
	MMP9	4318	2.94	2.06	1.85	9.79
	PCNA	5111	2.04	0.00	3.54	0.00
	PLK2	10769	11.54	0.00	3.63	0.00
	PLK3	1263	1.47	0.00	1.54	3.20
	PPM1D	8493	2.46	0.00	1.64	0.00
	TNFRSF10B	8795	2.33	0.00	2.46	0.00

Repressed Genes	BIRC5	332	0.63	16.09	0.43	3.52
	CCNB1	891	0.36	0.00	0.50	0.00
	HUS1	3364	0.30	16.09	0.81	16.52
	MDC1	9656	0.88	16.68	0.64	3.52
	MYC	4609	0.50	0.00	0.65	16.52

In our previous study [5], we identified 20 genes that were significant in both datasets using a permutation test. It was found that 15 genes out of 20 were re-identified in this study, with 5 genes (BTG2, CDKN1A, MDM2, MR1, and XPC) excluded in SAM analysis. To minimize the possibility of excluding important genes, we combined the two gene lists identified in the SAM analysis and the permutation test, resulting in a list of 26 genes. Note that all 26 genes are in the list of 221 genes found in our literature review [5].

### Pathway analysis results

**Figure 1:**
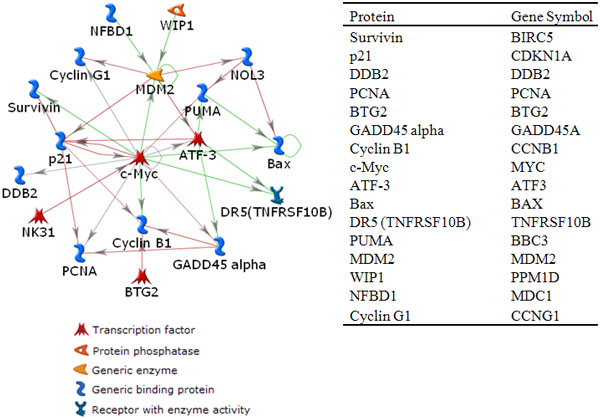
**A directly connected protein interaction network**. This protein interaction network was obtained when 26 genes were entered into MetaCore software. This network consists of 16 nodes and 28 edges. The MYC gene has 12 connections. The table on the right shows corresponding gene symbols for proteins in the network. Red, green, and gray lines indicate inhibitory, stimulatory, and unspecified interactions, respectively.

These 26 genes were fed into MetaCore software to identify a key interacting network. Figure 1 illustrates a directly connected protein-protein interaction network produced by MetaCore. This network consisted of 16 directly connected nodes and 28 edges. For the remaining 10 genes, there was no single connection. In this network, the MYC gene seems to play a key role as a hub gene with 12 connections. We assume that this network is reliable, considering it as a gold-standard network to assess the accuracy of the GRNs estimated by the graphical lasso algorithm. Due to the static nature of the microarray datasets and the unidirectional property of GRNs resulting from the graphical lasso algorithm, we removed the directionality from the network in Figure 1 to ease the comparison between the unidirectional gold-standard network and the estimated GRNs.

### Identification of gene regulatory networks

For the 16 genes shown in Figure 1, expression measurements after irradiation were extracted from the GSE1977 and GSE23393 microarray datasets and were used in the graphical lasso algorithm. Using different  λ values, a large range of candidate networks were yielded. Table 3 summarizes our experimental results. Table 3**(A) **and **(B) **show comparison results of networks estimated from GSE1977 and GSE23393 datasets, respectively, with the gold-standard network. To calculate a *p*-value of a result obtained for each  λ in Table 3, a simulation was performed. For example, using $-\text{lo}{\text{g}}_{10}\left(\lambda \right)=1.2$ in GSE1977, the estimated network had 27 edges with precision = 0.41, recall = 0.39, and *f*-score = 0.4. The common number of edges between the estimated network and the gold-standard network was 11. For the simulation, we randomly created two networks, both having 16 nodes: one with 28 edges (the number of edges in the gold-standard network) and the other with 27 edges, as in the estimated network. Then, a *f*-score was calculated between the two networks. This procedure was repeated 10,000 times. From our simulation results, the number of times that the *f*-score was larger than 0.4 (note that the *f*-score in the above example was 0.4) was 50. Therefore, its *p*-value was 50/10000 = 0.005. As shown in Table 3, the networks estimated from GSE1977 had larger *f*-scores than those estimated from GSE23393 and overall, their *p*-values were statistically significant.

**Table 3:** Results obtained using the graphical lasso algorithm.

$-\text{lo}{\text{g}}_{10}\left(\lambda \right)$	TP	FN	FP	# of edges	Precision	Recall	*f*-score	*p-*value
0.80	4	24	4	8	0.50	0.14	0.22	0.055
0.96	5	23	7	12	0.42	0.18	0.25	0.062
1.05	8	20	11	19	0.42	0.29	0.34	0.017
1.10	10	18	15	25	0.40	0.36	0.38	0.007
1.20	11	17	16	27	0.41	0.39	0.40	0.005
1.31	12	16	20	32	0.38	0.43	0.40	0.010
1.48	15	13	23	38	0.39	0.54	0.45	0.001
1.52	16	12	25	41	0.39	0.57	0.46	0.001
1.72	17	11	32	49	0.35	0.61	0.44	0.002
1.78	17	11	36	53	0.32	0.61	0.42	0.007
(A) Comparison of the gold-standard network and networks estimated from GSE1977,								

$-\text{lo}{\text{g}}_{10}\left(\lambda \right)$	**TP**	**FN**	**FP**	**# of edges**	**Precision**	**Recall**	***f*-score**	***p-*value**

0.55	1	27	5	6	0.17	0.04	0.06	0.737
0.58	2	26	8	10	0.20	0.07	0.11	0.596
0.71	3	25	12	15	0.20	0.11	0.14	0.599
0.80	5	23	17	22	0.23	0.18	0.20	0.420
1.04	7	21	23	30	0.23	0.25	0.24	0.337
1.13	9	19	26	35	0.26	0.32	0.29	0.194
1.15	10	18	27	37	0.27	0.36	0.31	0.150
1.31	12	16	32	44	0.27	0.43	0.33	0.110
1.39	13	15	35	48	0.27	0.46	0.34	0.096
1.46	13	15	38	51	0.25	0.46	0.33	0.135
(B) comparison of the gold-standard network and networks estimated from GSE23393, and								

$-\text{lo}{\text{g}}_{10}\left(\lambda \right)$**in GSE1977**	$-\text{lo}{\text{g}}_{10}\left(\lambda \right)$**in GSE23393**		**# of edges in GSE1977**		**# of edges in GSE23393**	**# of common edges**	***f*-score**	***p-value***

0.80	0.55		8		6	1	0.14	0.324
0.96	0.58		12		10	2	0.18	0.228
1.05	0.71		19		15	4	0.24	0.123
1.10	0.80		25		22	8	0.34	0.022
1.20	1.04		27		30	12	0.42	0.004
1.31	1.13		32		35	17	0.51	< 0.001
1.48	1.15		38		37	19	0.51	< 0.001
1.52	1.31		41		44	22	0.52	0.001
1.72	1.39		49		48	26	0.54	0.001
1.78	1.46		53		51	29	0.56	<0.001
(C) comparison of networks estimated from GSE1977 and GSE23393.								

**Figure 2:**
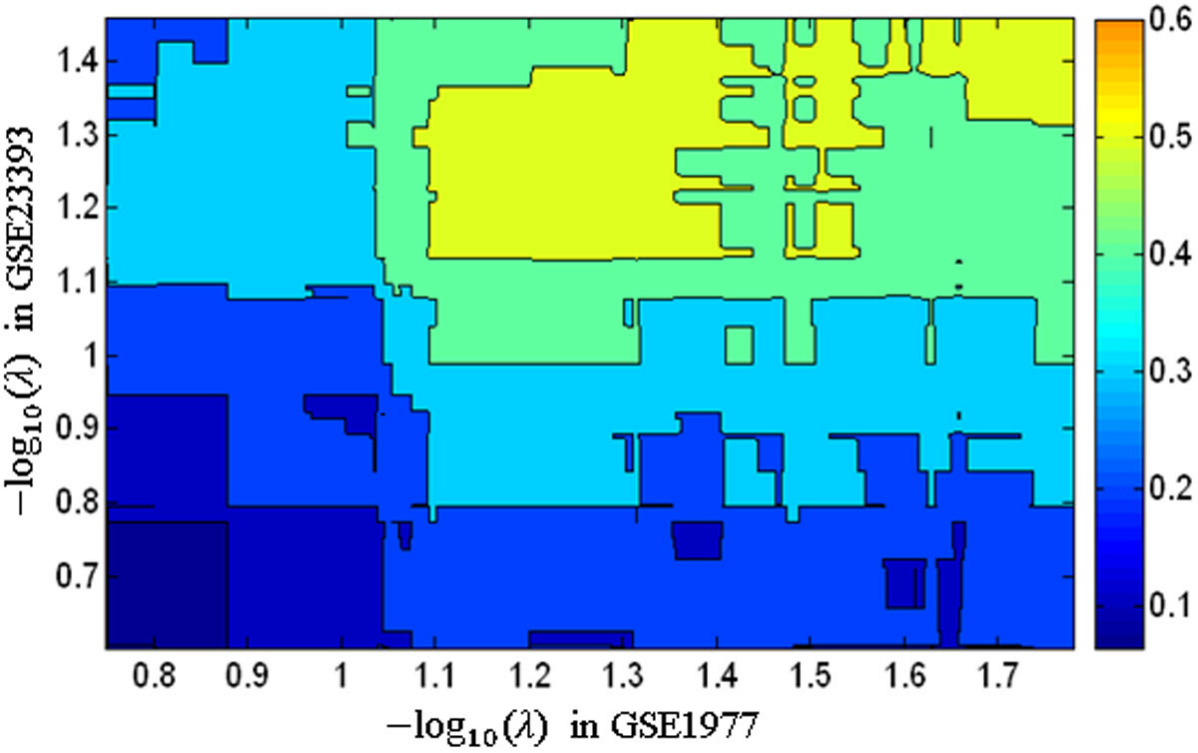
**Accuracy of estimated networks**. The *f*-scores calculated from two networks estimated using GSE1977 and GSE23393 with different  λ values.

**Figure 3:**
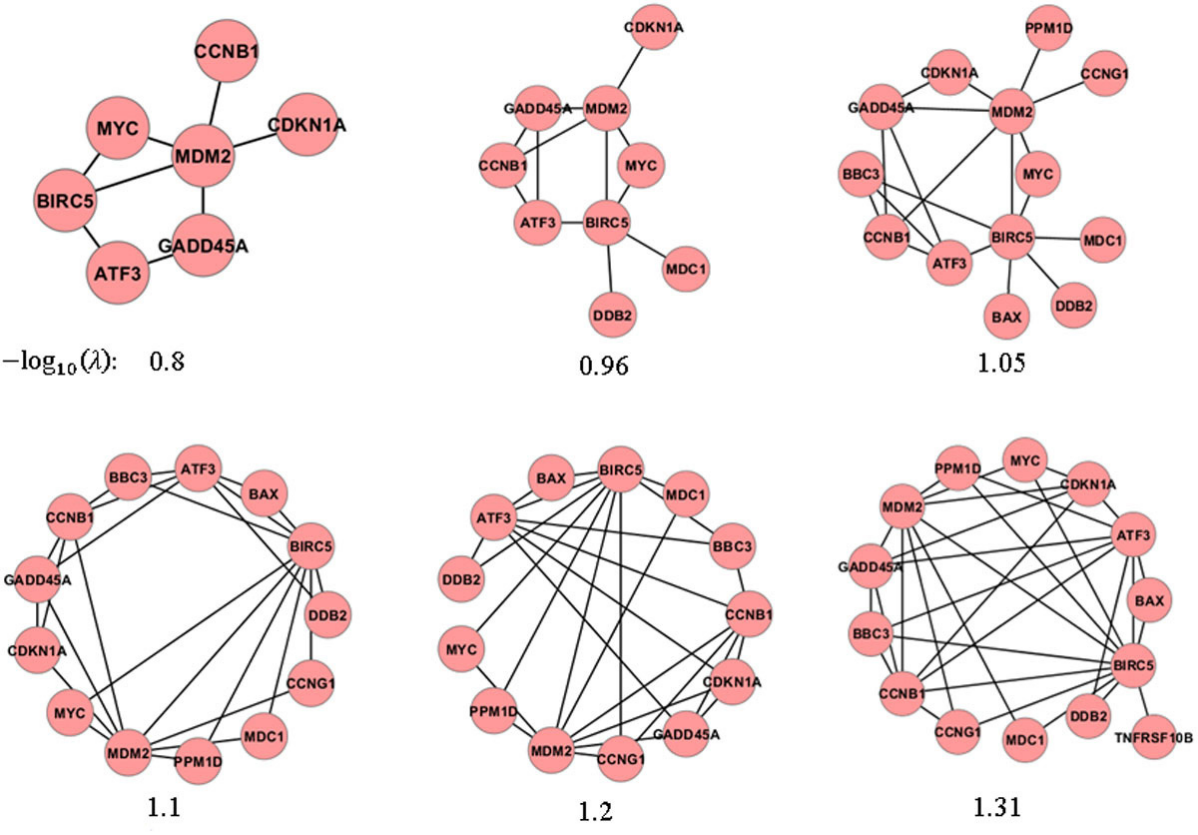
**Change in estimated networks**. Networks estimated when the graphical lasso algorithm was applied to GSE1977 with 6 different  λ values.

Table 3**(C) **shows results of the comparison between the networks estimated from GSE1977 and GSE23393 datasets. We note that the *f*-scores were greater than those between the gold-standard network and the networks estimated using GSE1977 or GSE23393. Their *p*-values were statistically significant when $-\text{lo}{\text{g}}_{10}\left(\lambda \right)\geq 1.1$ in GSE1977 and $-\text{lo}{\text{g}}_{10}\left(\lambda \right)\geq 0.8$ in GSE23393. However, as $-\text{lo}{\text{g}}_{10}\left(\lambda \right)$ values increased, the complexity of models (the number of edges in estimated networks) also increased. We compared the gold-standard network with estimated networks that have the number of edges that was similar to the gold-standard network (the number of edges: 28). In comparison between the gold-standard network and networks estimated from GSE1977 with $-\text{lo}{\text{g}}_{10}\left(\lambda \right)=1.2,$ the number of edges was 27, *f*-score was 0.4, and *p*-value was 0.005. In comparison between the gold-standard network and networks estimated from GSE23393 with $-\text{lo}{\text{g}}_{10}\left(\lambda \right)=1.04,$ the number of edges was 30, *f*-score was 0.24, and *p*-value was 0.337. In a comparison of the two networks estimated using GSE1977 (with $-\text{lo}{\text{g}}_{10}\left(\lambda \right)=1.2$) and GSE23393 (with $-\text{lo}{\text{g}}_{10}\left(\lambda \right)=1.04$), the *f*-score was 0.42 and *p*-value was 0.004. Figure 2 shows the change in *f*-scores calculated from two networks estimated using GSE1977 and GSE23393 with different  λ values. Figure 3 illustrates the change in networks estimated when the graphical lasso algorithm was applied to GSE1977 with 6 different  λ values.

## Discussion

To identify radio-responsive GRNs, we employed the graphical lasso algorithm using a list of radio-responsive genes selected from literature review and microarray data analysis. For the identification of significant genes with two publicly available microarray datasets (GSE1977 and GSE23393), we used SAM analysis. As a result, we found 21 genes to be important with FDR < 20%. These 21 genes included 15 of 20 genes that we identified in our previous study using a permutation test. This result suggests that we may miss important genes by using only a single statistical approach. In this study, we combined the two gene sets for further analysis, resulting in a list of 26 genes, including genes related to DNA repair: GADD45A, XPC, DDB2, and PCNA [2].

These 26 genes were entered into MetaCore software for a systems biology analysis. We identified a protein-protein interaction network that was used as a gold-standard network in this study. This network consisted of 16 nodes and 28 edges. Interestingly, the MYC gene had 12 connections, implying that this gene may play an important role in DNA-damage-related biological functions. It has been known that MYC is a key regulator of cell proliferation and apoptosis [16-19]. However, the role of MYC is not fully understood, due to its contradictory effect in enhancing or reducing radioresponsiveness [20,21].

The *f*-scores calculated to compare the gold-standard network with the networks estimated from GSE1977 were larger than those found in comparing the gold-standard network with the networks estimated from GSE23393. It was noted that the *f*-scores calculated from the two networks estimated from GSE1977 and GSE23393 were larger than those calculated from the gold-standard network and the networks estimated from GSE1977. This means that there are some common edges between the two networks estimated from GSE1977 and GSE23393, which are not present in the gold-standard network, suggesting that these edges could represent unknown associations between the genes.

In this study, we compared the estimated networks with a gold-standard network to investigate the change in the accuracy and number of interactions between genes with different  λ values. However, in general, one needs to find the best regularization parameter  λ, taking into account a tradeoff between model prediction and model complexity [10,22]. Bayesian information criterion (BIC) and Akaike criterion (AIC) are widely used for model selection.

Despite the lack of available datasets regarding radiation response, we demonstrated the presented methodology has the potential to identify radio-responsive GRNs via the graphical lasso algorithm based on literature review and microarray data analysis.

## Conclusions

We demonstrated that the graphical lasso algorithm can be a useful tool to reconstruct GRNs. We used a biomarker filtering method proposed in our previous study based on literature review and microarray data analysis. To evaluate the accuracy of radio-responsive GRNs estimated from two publicly available microarray datasets, we used a reliable protein interaction network generated from a curated database as a gold-standard network. It is expected that our proposed method can be efficiently used to identify not only significant radio-responsive genes, but also radio-responsive GRNs.

## Competing interests

The authors declare that they have no competing interests.

## Authors' contributions

JHO performed data analysis and wrote the manuscript. JOD supervised the study and edited the paper. Both authors read and approved the final manuscript.

## References

[B1] Templin T Paul S Amundson SA Young EF Barker CA Wolden SL Smilenov LB Radiation-induced micro-RNA expression changes in peripheral blood cells of radiotherapy patients Int J Radiat Oncol Biol Phys 2011 80 2 549 557 21420249 10.1016/j.ijrobp.2010.12.061 PMC3589812

[B2] Rieger KE Chu G Portrait of transcriptional responses to ultraviolet and ionizing radiation in human cells Nucleic Acids Res 2004 32 16 4786 4803 15356296 10.1093/nar/gkh783 PMC519099

[B3] Rieger KE Hong WJ Tusher VG Tang J Tibshirani R Chu G Toxicity from radiation therapy associated with abnormal transcriptional responses to DNA damage Proc Natl Acad Sci USA 2004 101 17 6635 6640 15096622 10.1073/pnas.0307761101 PMC404097

[B4] Eschrich S Zhang H Zhao H Boulware D Lee JH Bloom G Torres-Roca JF Systems biology modeling of the radiation sensitivity network: a biomarker discovery platform Int J Radiat Oncol Biol Phys 2009 75 2 497 505 19735874 10.1016/j.ijrobp.2009.05.056 PMC2762403

[B5] Oh JH Wong HP Wang X Deasy JO A bioinformatics filtering strategy for identifying radiation response biomarker candidates PLoS One 2012 7 6 e38870 22768051 10.1371/journal.pone.0038870 PMC3387230

[B6] Vignes M Vandel J Allouche D Ramadan-Alban N Cierco-Ayrolles C Schiex T Mangin B de Givry S Gene regulatory network reconstruction using Bayesian networks, the Dantzig Selector, the Lasso and their meta-analysis PLoS One 2011 6 12 e29165 22216195 10.1371/journal.pone.0029165 PMC3246469

[B7] Brouard C Vrain C Dubois J Castel D Debily MA d'Alché-Buc F Learning a Markov Logic network for supervised gene regulatory network inference BMC Bioinformatics 2013 14 1 273 24028533 10.1186/1471-2105-14-273 PMC3849013

[B8] Cai X Bazerque JA Giannakis GB Inference of gene regulatory networks with sparse structural equation models exploiting genetic perturbations PLoS Comput Biol 2013 9 5 e1003068 23717196 10.1371/journal.pcbi.1003068 PMC3662697

[B9] Aijö T Lähdesmäki H Learning gene regulatory networks from gene expression measurements using non-parametric molecular kinetics Bioinformatics 2009 25 22 2937 2944 19706742 10.1093/bioinformatics/btp511

[B10] Menéndez P Kourmpetis YA ter Braak CJ van Eeuwijk FA Gene regulatory networks from multifactorial perturbations using Graphical Lasso: application to the DREAM4 challenge PLoS One 2010 5 12 e14147 21188141 10.1371/journal.pone.0014147 PMC3004794

[B11] Friedman J Hastie T Tibshirani R Sparse inverse covariance estimation with the graphical lasso Biostatistics 2008 9 3 432 441 18079126 10.1093/biostatistics/kxm045 PMC3019769

[B12] Tusher VG Tibshirani R Chu G Significance analysis of microarrays applied to the ionizing radiation response Proc Natl Acad Sci USA 2001 98 9 5116 5121 11309499 10.1073/pnas.091062498 PMC33173

[B13] Sun H Li H Robust Gaussian graphical modeling via l1 penalization Biometrics 2012 68 4 1197 1206 23020775 10.1111/j.1541-0420.2012.01785.x PMC3535542

[B14] Bien J Tibshirani RJ Sparse estimation of a covariance matrix Biometrika 2011 98 4 807 820 23049130 10.1093/biomet/asr054 PMC3413177

[B15] Chiam TC Cho YR Accuracy improvement in protein complex prediction from protein interaction networks by refining cluster overlaps Proteome Sci 2012 10 Suppl 1 S3 22759580 10.1186/1477-5956-10-S1-S3 PMC3380738

[B16] Desbarats L Schneider A Müller D Bürgin A Eilers M Myc: a single gene controls both proliferation and apoptosis in mammalian cells Experientia 1996 52 12 1123 1129 8988255 10.1007/BF01952111

[B17] Vafa O Wade M Kern S Beeche M Pandita TK Hampton GM Wahl GM c-Myc can induce DNA damage, increase reactive oxygen species, and mitigate p53 function: a mechanism for oncogene-induced genetic instability Mol Cell 2002 9 5 1031 1044 12049739 10.1016/s1097-2765(02)00520-8

[B18] Kerr JF Winterford CM Harmon BV Apoptosis. Its significance in cancer and cancer therapy Cancer 1994 73 8 2013 2026 8156506 10.1002/1097-0142(19940415)73:8<2013::aid-cncr2820730802>3.0.co;2-j

[B19] Hermeking H Eick D Mediation of c-Myc-induced apoptosis by p53 Science 1994 265 5181 2091 2093 8091232 10.1126/science.8091232

[B20] Chiang CS Sawyers CL Mcbride WH Oncogene Expression and Cellular Radiation Resistance: A Modulatory Role for c-myc Mol Diagn 1998 3 1 21 27 10096954 10.154/MODI00300021

[B21] Gatti G Maresca G Natoli M Florenzano F Nicolin A Felsani A D'Agnano I MYC prevents apoptosis and enhances endoreduplication induced by paclitaxel PLoS One 2009 4 5 e5442 19421315 10.1371/journal.pone.0005442 PMC2673584

[B22] Wang Z Xu W San Lucas FA Liu Y Incorporating prior knowledge into Gene Network Study Bioinformatics 2013 29 20 2633 2640 23956306 10.1093/bioinformatics/btt443 PMC3789546

